# *NOD2* gene variants confer risk for secondary sclerosing cholangitis in critically ill patients

**DOI:** 10.1038/s41598-017-06268-y

**Published:** 2017-08-01

**Authors:** Christoph Jüngst, Vanessa Stadlbauer, Matthias C. Reichert, Vincent Zimmer, Susanne N. Weber, Lisa Ofner-Ziegenfuß, Torsten Voigtländer, Walter Spindelböck, Peter Fickert, Gabriele I. Kirchner, Frank Lammert, Tim O. Lankisch, Marcin Krawczyk

**Affiliations:** 1 Department of Medicine II, Saarland University Medical Center, Saarland University, Homburg, Germany; 2 Division of Gastroenterology and Hepatology, Medical University of Graz, Graz, Austria; 3 Institute of Human Genetics, Medical University of Graz, Graz, Austria; 4 Department of Gastroenterology, Hepatology and Endocrinology, Hannover Medical School, Hannover, Germany; 5 Department of Internal Medicine I, University of Regensburg, Regensburg, Germany

## Abstract

Sclerosing cholangitis in critically ill patients (SC-CIP) is a progressive cholestatic disease of unknown aetiology characterized by chronic biliary infections. Hence we hypothesized that common *NOD2* (nucleotide-binding oligomerisation domain containing 2) gene variants, known risk factors for Crohn’s disease and bacterial translocation in liver cirrhosis, increase the odds of developing SC-CIP. Screening of 4,641 endoscopic retrograde cholangiography procedures identified 17 patients with SC-CIP, who were then genotyped for the three common *NOD2* mutations (Cohort 1, discovery cohort). To validate the association, we subsequently tested these *NOD2* variants in 29 patients from SC-CIP cohorts of three additional medical centers (Cohort 2, replication cohort). In Cohort 1, the *NOD2* variants were present in 5 of 17 SC-CIP patients (29.4%), which is twice the frequency of the general population. These results were replicated in Cohort 2 with 8 patients (27.6%) showing *NOD2* mutations. In contrast, polymorphisms of hepatocanalicular transporter genes did not have major impact on SC-CIP risk. This first study on genetic susceptibility in SC-CIP patients shows an extraordinary high frequency of *NOD2* variation, pointing to a critical role of inherited impaired anti-bacterial defense in the development of this devastating biliary disease.

## Introduction

Secondary sclerosing cholangitis (SSC) is a chronic cholestatic disease potentially leading to cirrhosis, biliary infections, and decompensation of liver function^1^. Recently the development of secondary sclerosing cholangitis in critically ill patients (SC-CIP) has been recognized during or after intensive care unit (ICU) treatment^2^. These patients typically have no history of hepatobiliary disease and present with persistent cholestasis despite recovery from the primary illness. As the disease progresses, cholangiography shows irregular bile duct strictures and rarefications of small bile ducts, and additionally biliary casts may be detected by endoscopic retrograde cholangiopancreaticography (ERCP). The course of SC-CIP is often progressive and patients with SC-CIP might eventually require transplantation^2–6^. The specific pathomechanisms of SC-CIP are not understood, and initial ischemic injury of the biliary tree and subsequent biliary tract infections have been implicated^1, 6^. However, additional triggers as well as modulators of the disease course are suspected to play critical roles.

Nucleotide-binding oligomerization domain containing 2 (NOD2) is a component of the innate immune system and expressed, among others, in Paneth cells of the intestinal epithelium as well as hepatocytes, macrophages and dendritic cells^7–9^. After activation by the bacterial cell wall components muramyl dipeptide, NOD2 promotes nuclear factor NF-κB signaling and the induction of inflammatory mediators^10^. Three common *NOD2* gene mutations (p.R702W, p.G908R, c.3020insC) cause markedly reduced NF-κB activation^10^, thereby altering intestinal barrier functions and facilitating bacterial translocation. According to available data, the combined *NOD2* variant allele frequencies for all three variants were 6.9%^11^ and 7.8%^12^ in healthy adults. The mutations are known to confer an increased risk of Crohn’s disease^13, 14^. Moreover, in our previous study^15^ we demonstrated that the *NOD2* mutations increase the risk of developing spontaneous bacterial peritonitis and are associated with increased mortality in patients with liver cirrhosis.

It has been speculated that the formation of ‘toxic bile’ might contribute to the development of bile duct injury in SC-CIP^1^. Bile secretion is maintained by hepatocanalicular transport proteins, including the bile salt export pump (ABCB11), the phosphatidylcholine floppase ABCB4, and the phosphatidylserine flippase ATP8B1. Mutations in these genes cause progressive familial intrahepatic cholestasis, and transporter polymorphisms might potentially increase the risk for the development of other cholestatic liver diseases including SC-CIP. So far, genetic studies have not been carried out in SC-CIP.

Given these assumptions, we hypothesized that *NOD2* variants as well as selected variants in hepatocanalicular transporter genes might be associated with SC-CIP development. Therefore we conducted a pilot study, analyzing the most common *NOD2* mutations and selected variants of *ABCB4*, *ABCB11* and *ATP8B1* in our cohort of SC-CIP patients, and subsequently validated the findings in an independent cohort from three additional academic medical centers that had collected SC-CIP patients in the past decade.

## Results

### Clinical characteristics of the SC-CIP patients

The characteristics of Cohort 1 including data related to ICU treatment, clinical, endoscopic and laboratory data are presented in Table 1. The majority of patients were men (82.4%), and all patients reported Caucasian ethnicity. As for the cause of ICU admission almost half of the patients had myocardial infarction or cardiothoracic surgery. All patients required ventilation support and vasopressor therapy during their ICU treatment. ERCP showed typical cholangiographic findings of SSC and all of the patients displayed intrahepatic strictures and rarefications, whereas extrahepatic strictures were seen in two patients only. Endoscopic sphincterotomy was performed in all patients during ERCP. An endoscopically treatable post-sphincterotomy bleeding episode occured in one patient, which was the only adverse event. Endoscopic interventions (including biliary cast extraction, ballon dilation or intermittent stent placement) were performed in 14 patients (82.4%) during the ERCP and in two patients a temporary nasobiliary drainage was placed. Notably, all 17 SC-CIP patients had infectious complications, mainly sepsis (58.8%), during their stay on ICU. In six of eight patients with cholangitis, microbiological analysis of bile was performed, identifying *Entercoccus faecium* (66.7%) and *Enterococcus faecalis* (50%); one patient tested positive for *Candida* species. During the follow-up period, eight patients (47.1%) progressed to liver cirrhosis, and two of these needed liver transplantation. Liver-related laboratory parameters from the day of the first ERCP including γ-GT, AP, ALT, bilirubin and the MELD score were not correlated to the development of cirrhosis (data not shown). Due to a progressive course with persistent jaundice and infectious complications, three patients (17.7%) died within 7 to 15 months after diagnosis of SC-CIP. Overall these results confirm the dismal prognosis of SC-CIP. None of the patients had a history of inflammatory bowel disease.

**Table 1:** Clinical, endoscopic and laboratory characteristics of Cohort 1 comprising 17 patients with SC-CIP.

Parameter	Number
Age (years)	63 (33–80)
Gender	3 F/14 M
**Cause of ICU admission**	
Myocardial infarction	5 (29.4%)
Cardiothoracic surgery	3 (17.6%)
Polytrauma	3 (17.6%)
Pneumonia	3 (17.6%)
Acute pancreatitis	1 (5.9%)
Ruptured abdominal aortic aneurysm	1 (5.9%)
Major bleeding after thyroid surgery	1 (5.9%)
**ICU features**	
ICU days	32 (8–167)
Ventilation days	19 (4–147)
Vasopressors (%)	17 (100%)
Renal replacement therapy (%)	3 (17.6%)
Endoscopic features*	
Time from ICU admission to ERCP, days	105 (24–1155)
Intrahepatic strictures and rarefications	17/17 (100%)
Extrahepatic stricture	2/17 (11.7%)
Biliary casts	11/16 (68.8%)
Endoscopic interventions**	14/16 (87.5%)
Nasobiliary drainage	2/16 (12.5%)
Infectious complications***	
Sepsis	10 (58.8%)
Cholangitis	8 (47.0%)
Pneumonia	8 (47.0%)
Wound infection	2 (11.8%)
Abscess of the liver	1 (5.9%)
Colitis	1 (5.9%)
**Clinical course**	
Progression to liver cirrhosis	8 (47.1%)
Liver transplantation	2 (11.8%)
Death	3 (17.7%)
Laboratory values****	
ALT (U/l)	67 (20–1106)
Alkaline phosphatase (U/l)	428 (87–1305)
y-GT (U/l)	731 (135–1447)
Bilirubin (mg/dl)	3.45 (0.4–23.5)
Creatinine (mg/dl)	0.91 (0.6–9.4)
INR	1.02 (0.88–1.98)
CRP (mg/l)	28 (0.3–191)
MELD score (UNOS modified)	16 (6–22)
Follow-up (days)	467 (39–3535)

Values are given as medians and ranges.

Abbreviations: ALT, alanine aminotransferase; CRP, C-reactive protein; ERCP, endoscopic retrograde cholangiopancreaticography; F, female; γ-GT, gamma-glutamyl-transferase; ICU, intensive care unit; INR, international normalized ratio; M, male; MELD, model of end-stage liver disease; SC-CIP, sclerosing cholangitis in critically ill patients.

*In 16 of the 17 patients with SC-CIP in cohort 1, cholangiography was obtained by endoscopic retrograde cholangiography procedure, and in one patient magnet resonance cholangiography was performed only.

**Endoscopic interventions include biliary cast extraction, ballon dilation or intermittent stent placement.

***Patients may have had more than one infectious complication.

****Values from the day of the first ERCP.

Supplementary Table 1 summarizes the clinical characteristics of the 29 patients included in Cohort 2. Corresponding to Cohort 1, the majority of patients were men (75.7%) and received ventilation support and vasopressor therapy on ICU. In 25 patients ERCP was performed, showing mostly intrahepatic strictures, bile duct rarefications and biliary casts, whereas extrahepatic strictures were less common. Also infectious complications such as cholangitis and sepsis were highly prevalent. Seven patients (24.1%) received liver transplantation, and nine patients (31.0%) died.

### NOD2 mutations are frequent in patients with SC-CIP

**Figure 1:**
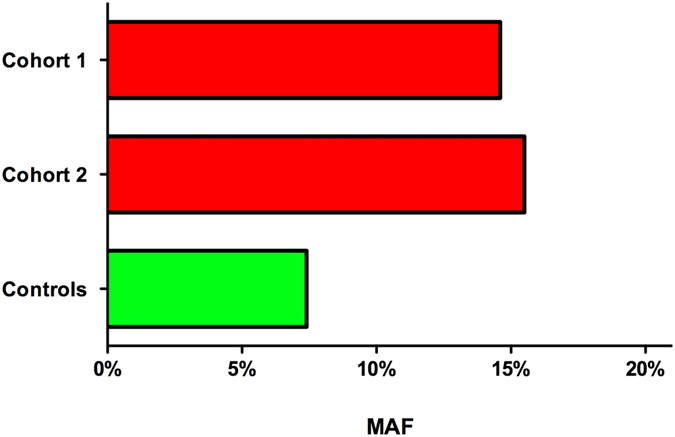
Distribution of *NOD2* risk allele frequencies in the two study cohorts and the general population as control (data of general population extracted from Hugot *et al*.^12^). MAF, minor allele frequency.

In Cohort 1 and Cohort 2 we detected markedly increased frequencies of the *NOD2* variants as compared to the general population (6.9–7.8%)^11, 12^. In our SC-CIP cohorts, the minor allele frequencies for all *NOD2* variants combined were 14.7% (Cohort 1) and 15.5% (Cohort 2), respectively (Fig. 1). In Cohort 1 we observed *NOD2* risk genotypes in 5 of the 17 SC-CIP patients (29.4%): four patients carried the p.R702W variant and one patient tested positive for the c.3020insC variant. In line with these findings, eight of 29 patients of Cohort 2 (27.6%) carried *NOD2* mutations: five patients were carriers of the p.R702W variant, two patients exhibited the p.G908R variant and one patient carried both, the p.R702W and the c.3020insC mutation, respectively. In both cohorts, all *NOD2* variants were present in the heterozygous state. Based on the published frequencies of the *NOD2* variants in healthy individuals^12^, we estimate that carriers of the *NOD2* minor alleles have at least a 2-fold increased risk of developing SC-CIP (OR = 2.19, 95% CI 1.16–2.95, p = 0.026).

Analysis of Cohort 1 revealed that four of the five patients with *NOD2* variants had multiple recurrent episodes of cholangitis whereas this was seen in only 33.3% of the *NOD2* wild-type patients (Table 2). However increased frequencies of the *NOD2* genotypes were not apparent in the SC-CIP patients with cholangitis in Cohort 2. Sepsis occurred in about 60% of the patients with *NOD2* variants and *NOD2* wild-type alleles, respectively. Three of the five patients with *NOD2* variants developed severe cirrhosis, and two of them underwent liver transplantation whereas deaths occurred only in three patients without *NOD2* variants. In Cohort 2, the presence of *NOD2* variants was not significantly related to the prevalence of infectious complications or survival (data not shown).

**Table 2:** *NOD2* genotypes and clinical course in SC-CIP patients (Cohort 1).

*NOD2* status	Wild-type	Variants*
Patients (%)	12/17 (70.6%)	5/17 (29.4%)
Cholangitis	4/12 (33.3%)	4/5 (80.0%)
Sepsis	7/12 (58.3%)	3/5 (60.0%)
Progression to cirrhosis	5/12 (41.7%)	3/5 (60.0%)
Liver transplantation	0	2/5 (40.0%)
Death	3/12 (25%)	0

*4 patients were carriers of the *NOD2* variant p.R702W, and one patient carried

the c.3020insC variant, all in heterozygous state.

Detailed results of the genotyping of hepatocanalicular transporter gene polymorphisms are presented in Supplementary Table 2. Overall, we did not detect any consistent effects of the tested variants on SC-CIP development or phenotypes, however we found a higher percentage of homozygous carriers of the *ABCB4* c.504 T > C variant in the SC-CIP patients in Cohort 2, but not in Cohort 1 as compared to the general population^11^.

## Discussion

This is the first genetic study performed in patients with SC-CIP. Our results are based on the analysis of thoroughly phenotyped patients from two independent cohorts and point to a critical role of *NOD2* genetic variation in the development of SC-CIP. Indeed, the prevalence of the *NOD2* risk variants in the SC-CIP patients was twice as high as in the general population^11, 12^, specifically the p.R702W variant was highly prevalent and well within the range of patients with Crohn’s disease^16, 17^. We hypothesize that ICU patients carrying *NOD2* variants might be predisposed to infectious complications, and hence may have an increased risk for the development of SSC in the setting of bile duct ischemia. This is in line with the observation that liver transplant recipients carrying the p.R702W variant are at increased risk of bacterial infections^18^. The distinct microbiological spectrum in bile, comprising predominantly *Entercoccus faecium* and *faecalis*, is consistent with previous reports^2, 19^. Interestingly, the frequency of *NOD2* variants in ICU patients with sepsis has been shown not to be increased compared to healthy controls^20^, but *NOD2* risk alleles, especially the c.3020insC variant, were associated with sepsis related mortality in these patients^20^. Therefore *NOD2* variants might confer an increased risk for a worse outcome in ICU patients depending on other co-factors, e.g. the development of SC-CIP in the case of coexisting ischemic injury to the biliary epithelium. Of note, in one experimental model, *NOD2* knock-out mice were protected from cholestatic liver injury by enhanced excretion of bile acids via the kidneys^21^. These results offer interesting perspectives for further research on pathophysiology of SSC in humans with *NOD2* variants. The studies by Karlsen *et al*.^22^ and Gaj *et al*.^23^ did not show an impact of *NOD2* variants on the development of primary sclerosing cholangitis (PSC) or primary biliary cholangitis (PBC), respectively. However, in contrast to these cholestatic diseases, apparent biliary infections are common in the early stages of SC-CIP, and the inflammatory reactions to an exogenous trigger might be modified by the genetic factor we identified. Hence, these results point to a specific risk of *NOD2* variants for SC-CIP and are in line with differences in the pathophysiology of SC-CIP as compared to PSC.

In contrast to the above results, genotyping of selected variants in the hepatobiliary transporter genes *ABCB4*, *ABCB11* and *ATP8B1* did not reveal major differences of genotypic distributions as compared to the general population. Potentially a more comprehensive approach by applying next generation sequencing might provide more insights on the impact of genetic variation in hepatobiliary transporters on the pathogenesis﻿ of SC-CIP.

Selective digestive tract decontamination (SDD) has recently been recommended in critically ill patients^24^ as intestinal bacteria have long been recognized as source of systemic infection. Studies with risk stratification according to the *NOD2* variant status might be of interest to see whether carriers of *NOD2* mutations particularly benefit from SDD, or non-antibiotic modulation of barrier function e.g. with bile acid derivates^25^. Given the low frequency and difficulty to early diagnose SC-CIP, it may only be speculated that these strategies or the non-absorbable antibiotic rifaximin would decrease the incidence of SC-CIP in at-risk patients.

In addition to the increased risk for bacterial translocation and subsequent infection, *NOD2* variants might also impact regulation of inflammatory processes in the hepatobiliary system. *NOD2* expression has been detected in hepatocytes and inflammatory cells of the liver, and NOD2 was able to stimulate cellular immune mechanisms in response to muramyl dipeptide^7^. Correspondingly, Scott *et al*.^9^ described NOD2 in hepatocytes as part of an immune network responding to pathogens. Recently inflammatory responses secondary to bacterial infections and endoplasmatic reticulum stress were shown to be mediated through a NOD1- and NOD2-dependent pathway that could be blunted by tauroursodeoxycholic acid^26^. Another study demonstrated that defective NOD2 sensing increases intestinal translocation and bacterial invasion in metabolic tissues, including the liver, leading to inflammation and insulin resistance^27^. In addition, intestinal dysbiosis in *NOD2* knock-out mice promoted inflammation and insulin resistance^27^. Cholangiocytes have been recognized as active players of the innate immune system, participating in the defense against bacteria by activation of inflammatory molecules^28^. However, potential effects of disturbed NOD2 sensing on biliary epithelial cells have not been defined so far.

One can argue, that small sample size might be a limitation of our study. However, due to the fact that SC-CIP is a very rare disease, it is evident that studies of the disease are restricted with respect to sample size. A careful analysis of a large data set allowed us to compile a reasonable size for Cohort 1 and by cooperation with other academic centers, we were able to replicate our results in Cohort 2.

In conclusion, in this first report on genetic risk factors for the development of SC-CIP, we could demonstrate a high prevalence of *NOD2* variants in independent patient cohorts, indicating that impaired bacterial defense mechanisms might play a critical role in SC-CIP development. These data are encouraging for more functional and microbial studies to fully define the role of a deficient NOD2 sensing system in chronic inflammatory hepatobiliary diseases in general and SC-CIP in particular.

## Patients and Methods

### SC-CIP cohorts

The discovery cohort (Cohort 1) was collected by screening data of 4,641 ERCP procedures performed between January 2008 and April 2015 in the Department of Medicine II at Saarland University Medical Center as well as patient records of this time period. In total, we identified 17 patients with a history of ICU treatment complicated by persistent elevation of laboratory markers of cholestasis and cholangiographic signs of SSC (for details see Table 1). None of these patients had evidence of preexisting hepatobiliary disease prior to ICU admission. The diagnosis of SC-CIP was based on typical cholangiographic criteria such as irregular intrahepatic bile ducts with strictures and prestenotic dilations as well as bile duct rarefications or biliary casts. In 16 of the 17 patients cholangiography was performed by ERCP, and in one patient characteristic signs of SSC were depicted by magnetic resonance cholangiography (MRCP) only. No pathologies in liver arteries, portal and hepatic veins in duplex ultrasound and/or magnetic resonance imaging were detected in any of the patients. The diagnosis of infectious cholangitis was based on the presence of the following criteria: jaundice, fever/chills, abdominal pain, laboratory markers of systemic inflammation, and detection of bacteria in bile collected during ERCP in the majority of patients.

Furthermore, by cooperation with three other university centers with dedicated interest and expertise in SSC (one in Austria and two in Germany), we collected an independent group of 29 SC-CIP patients (Cohort 2) for replication. Cohort 2 comprised 12 patients from a previously published SC-CIP cohort^19^ from Hannover Medical School (Germany), 10 patients collected at the Medical University of Graz (Austria), and seven patients from a SC-CIP cohort at the University Hospital Regensburg (Germany). The study was conducted according to the Declaration of Helsinki and approved by the ethic committee of Ärztekammer des Saarlandes (271/11). All patients in the study provided informed consent.

### Data collection

Relevant clinical data was collected by chart review, including indication for ICU admission, use of vasopressors and mechanical ventilation, need for renal replacement therapy, infectious complications, and antibiotic treatment. Laboratory tests were reviewed and the model of end-stage liver disease (MELD) scores were calculated for each patient at the day of the first ERCP or MRCP. Follow-up information was compiled to analyze the outcome of the patients.

### Endoscopic procedures

SSC was diagnosed by ERCP in most cases, applying the endoscopic criteria specified above in the patient cohort section. For microbiological analysis, bile was collected during cannulation of the common bile duct. Biliary casts were extracted with endoscopic balloons or extraction baskets; dilation of stenosis and intermittent stent placement was carried out as considered necessary. After placement of a nasobiliary drainage in two patients of Cohort 1, continuous flushing of the bile ducts with saline solution was conducted for at least three days.

### Genotyping

After extraction of genomic DNA from EDTA-anticoagulated blood using a membrane-based extraction kit (Qiagen, Hilden, Germany), genotyping of the three common *NOD2* gene variants (*rs2066844* [p.R702W], *rs2066845* [p.G908R], *rs2066847* [c.3020insC]) was performed as described^15^.

Variants of the hepatocanalicular transporter genes *ABCB4* (*rs1202283* [c.504 T > C], *rs2109505* [c.711 A > T], *rs45575636* [p.R590Q]), *ABCB11* (*rs2287622* [p.A444V], *rs497692* [c.3084 A > G], *rs72549402* [p.D482G]) and *ATP8B1* (*rs12968116* [p.R952Q], *rs121909100* [p.I661T]) as well as the farnesoid X receptor *NR1H4* (*rs56163822* [c.−1g > t]), which encodes the central bile acid sensor^29^, were genotyped by 5′-nuclease PCR based assay with allele specific fluorescent probes (Fisher Scientific, Schwerte, Germany) on a TaqMan^®^ Real Time PCR Fast 7500 system (Applied Biosystems, Foster City, CA, USA). For genotyping of the *ATP8B1* variant *rs34018205* (p.E429A), the following self-designed primers and probes were used: forward TCAACTGGGACCTGCAAATGT, VIC-TGTCCTTC**T**CAGCATAGT, FAM-CCTTC**G**CAGCATAGT.

In brief, 10 µl PCR reactions contained 20 ng genomic DNA, 1x TaqMan GTXpress Master Mix (Applied Biosystems), 900 nM of each primer, and 200 nM of VIC-labeled and FAM-labeled probes, respectively. Amplification conditions consisted of 95 °C for 20 seconds, followed by 40 cycles of 95 °C for 3 seconds and 60 °C for 30 seconds.

The variants *ABCB11 rs11568372* (p.E297G) and *ATP8B1 rs146599962* (p.N45T) were genotyped by direct sequencing with the Big Dye^®^ Terminator 1.1 sequencing kit (Applied Biosystems) of 268 and 295 bp amplicons, respectively, containing the mutations of interest. The following primers were used: *ABCB11* forward TGTGTCCAAGTTTACGGACTATGA, reverse TACTCTGCTTAGCTCCCTCTT; *ATP8B1* forward TGCAGGCAGTATTCAACCAA. The annealing temperature was 60 °C.

### Statistical analysis

Quantitative variables were expressed as median and ranges. Exact tests were performed to check the consistency of genotyping results with Hardy-Weinberg equilibrium (http://ihg.gsf.de/cgi-bin/hw/hwa1.pl). Differences of allele and genotype frequencies were assessed by *x* ^2^ tests (http://ihg.gsf.de/cgi-bin/hw/hwa1.pl).

### Data availability

The datasets generated during and/or analysed during the current study are available from the corresponding author on reasonable request.

## Electronic supplementary material

Supplementary Information
