# Supplementary material for: NOD2 gene variants confer risk for secondary sclerosing cholangitis in critically ill patients
Source: Sci Rep. 2017 Aug 1;7:7026. doi: 10.1038/s41598-017-06268-y (PMC5539147; doi:10.1038/s41598-017-06268-y)
Supplement: Supplementary file 1 — Supplementary Information [file 41598_2017_6268_MOESM1_ESM.pdf]

Supplementary material

NOD2 gene variants confer risk for secondary sclerosing cholangitis in critically ill patients

Christoph Jüngst[1*], Vanessa Stadlbauer[2*], Matthias C Reichert[1], Vincent Zimmer[1], Susanne N Weber[1], Lisa Ofner-Ziegenfuß[3], Torsten Voigtländer[4], Walter Spindelböck[2], Peter Fickert[2], Gabriele I Kirchner[5], Frank Lammert[1#], Tim O Lankisch[4#] and Marcin Krawczyk[1#]

Supplementary table 1: Clinical, endoscopic and laboratory characteristics of the replication cohort (Cohort 2) comprising 29 patients with SC-CIP

**Parameter**	**Number**
Age (years)	54 (16 - 74)
Gender	7 F/22 M
**ICU features**	
ICU days	43 (14 - 229)
Ventilation days	30 (9 - 165)
Vasopressors (%)	25 (86.2%)
Renal replacement therapy (%)	10 (34.5%)
**Endoscopic features**[#]	
Time from ICU admission to ERCP/MRCP, days	85 (8 - 1403)
Intrahepatic strictures and rarefications	23/27 (85.2%)
Extrahepatic strictures	6/27 (22.2%)
Biliary casts	22/25 (88.0%)
Nasobiliary drainage	0
**Clinical features**	
Sepsis	22 (75.9%)
Cholangitis	16 (55.2%)

Progression to liver cirrhosis	9 (31.0%)
Liver transplantation	7 (24.1%)
Death	9 (31.0%)
**Laboratory values***	
ALT (U/l)	117 (40 - 361)
Alkaline phosphatase (U/l)	884 (140 - 3366)
y-GT (U/l)	939 (113 - 2999)
Bilirubin (mg/dl)	4.69 (0.50 - 35.51)
Creatinine (mg/dl)	1.0 (0.41 - 3.33)
INR	1.15 (0.90 - 2.38)
CRP (mg/l)	36 (0.6 - 195)
MELD score (UNOS modified)	15 (6 - 36)
Follow-up (days)	748 (5 - 3966)

Values are given as medians and ranges.

Abbreviations: ALT, alanine aminotransferase; CRP, C-reactive protein; ERCP, endoscopic retrograde cholangiopancreaticography; F, female; γ-GT, gamma-glutamyl-transferase; ICU, intensive care unit; INR, international normalized ratio; M, male; MELD, model of end-stage liver disease; MRCP, magnetic resonance cholangiopancreaticography, SC-CIP, sclerosing cholangitis in critically ill patients.

[#]In 25 of the patients with SC-CIP, cholangiography was obtained by endoscopic retrograde cholangiography procedure, and in 2 patients magnet resonance cholangiography was

performed only. In 2 patients SC-CIP was diagnosed by typical clinical and histological features plus ultrasound, respectively.

*Values from the day of the first ERCP or MRCP.

Supplementary table 2: Genotype distribution of selected variants in the hepatobiliary transporter genes *ABCB4*, *ABCB11* and *ATP8B1* of the SC-CIP patients of Cohort 1 and 2

Polymorphism	Cohort 1 n = 17 (%)	Cohort 2 n = 28 (%)
**ABCB4**		
c.504T>C (rs1202283)		
CC	2 (11.8)	3 (10.7)
TC	9 (52.9)	9 (32.1)
TT	6 (35.3)	16 (57.1)
c.711A>T (rs2109505)		
TT	1 (5.9)	0
AT	6 (35.3)	5 (17.9)
AA	10 (58.8)	23 (82.1)
**ABCB11**		
p.A444V (rs2287622)		
TT	4 (23.5)	3 (10.7)
CT	6 (35.3)	15 (53.6)
CC	7 (41.2)	10 (35.7)
c.3084A>G (rs497692)		
AA	2 (11.8)	5 (17.9)
AG	10 (58.8)	20 (71.4)
GG	5 (29.4)	3 (10.7)

ATP8B1

p.R952Q (rs12968116)

GG	12 (70.6)	22 (78.5)
GA	5 (29.4)	5 (17.9)
AA	0	1 (3.6)

None of the patients of Cohort 1 and Cohort 2 carried the following variants in the hepatocanalicular transporter genes: *ABCB4* (rs45575636 [p.R590Q]), *ABCB11* (*rs11568372* [p.E297G], *rs72549402* [p.D482G]), and *ATP8B1* (*rs146599962* [p.N45T], *rs34018205* [p.E429A], *rs121909100* [p.I661T]). One patient of Cohort 1 was positive for the variant (*rs56163822* [c.-1g>t]) in the farnesoid X receptor *NR1H4*. For Cohort 2, blood samples from 28 of the 29 patients were available for genetic analysis of the hepatocanalicular transporter genes.
